# CHOLANGIOCARCINOMA: EPIDEMIOLOGY, HISTOPATHOLOGY, AND POTENTIAL PROGNOSTIC AND THERAPEUTIC IMPLICATIONS IN A COHORT FROM A REFERENCE CENTER IN SOUTHERN BRAZIL

**DOI:** 10.1590/0102-6720202400057e1851

**Published:** 2025-01-13

**Authors:** João Pedro Pattussi BERTINATTI, Josenel Maria Barcelos MARÇAL, Eduardo CAMBRUZZI, Dido Eliphas LEÃO DE ALENCAR

**Affiliations:** 1 Santa Casa de Misericórdia de Porto Alegre – Porto Alegre (RS), Brazil.

**Keywords:** Bile duct diseases, Cholangiocarcinoma, Epidemiology, Pathology, Prognosis, Doenças dos ductos biliares, Colangiocarcinoma, Epidemiologia, Patologia, Prognóstico

## Abstract

**BACKGROUND:**

Cholangiocarcinoma (CCA) is a rare neoplasm, with high mortality, originating in the bile ducts. Its incidence is higher in Eastern countries due to the endemic prevalence of liver parasites. Factors such as metabolic syndrome, smoking, and pro-inflammatory conditions are also linked to the disease. Clinical features include asthenia, abdominal pain, cholestasis, and increased serum levels of CEA and CA19-9.

**AIMS:**

The aim of this study was to evaluate CCA prevalence, survival, and potential prognostic and therapeutic implications in a patient cohort and assess correlations with clinical laboratory data and possible associated risk factors.

**METHODS:**

This is a retrospective study of the clinical and histological data of patients diagnosed with CCA at Santa Casa de Misericórdia in Porto Alegre, Brazil, between 2016 and 2021.

**RESULTS:**

There was a 56% prevalence of CCA in women, with intrahepatic localization in 55.4% of cases and unifocality in 85.6% of patients. The mean age of the patients was 63 years (26–89 years), with a mean tumor size of 5.5 cm. The median survival time was 7 months (0 to >50). CA19-9 was altered in 81% of patients, whereas GOT/GPT was altered in 62.5% and gamma-glutamyl transferase/alkaline phosphatase/bilirubin in 69.1% of patients. Mortality was higher among patients with extrahepatic CCA.

**CONCLUSION:**

Risk factors such as smoking, cholecystectomy, cirrhosis, intrahepatic lithiasis, and transplantation should be considered individually by the attending physician for radiological monitoring and incidental discovery of the neoplasm. Lack of timely identification by the attending physician can delay diagnosis, increasing mortality.

## RESUMO

**RACIONAL:**

Os colangiocarcinomas (CCA) são neoplasias raras, de elevada mortalidade, originadas nas vias biliares. Sua incidência é maior no oriente devido à endemia de parasitas hepáticos. Fatores como a síndrome metabólica, tabagismo e causas pró-inflamatórios também estão associados. A clínica desta entidade caracteriza-se por astenia, dor abdominal, colestase e aumento dos níveis séricos de CEA e CA19-9.

**OBJETIVOS:**

Avaliar a prevalência, sobrevida e potenciais implicações prognósticas/terapêuticas em uma coorte de pacientes com CCA, correlacionando-se dados clínico-laboratoriais e possíveis fatores de risco associados.

**MÉTODOS:**

Análise retrospectiva por análise de dados clínicos e histológicos de pacientes diagnosticados com CCA na Santa Casa de Misericórdia de Porto Alegre, entre 2016 e 2021.

**RESULTADOS:**

Houve uma prevalência de 56% de colangiocarcinomas em mulheres, com localização intra-hepática em 55,4% dos casos e unifocalidade em 85,6% dos pacientes. A idade média dos pacientes foi de 63 (26-89) anos, com tamanho tumoral médio de 5,5 cm. O tempo de sobrevida mediano foi de 7 meses (0 a >50). O CA 19-9 estava alterando em 81% dos pacientes, enquanto TGO/TGP estavam alterados em 62,5% dos pacientes e Gama-GT/FA/bilirrubinas em 69,1%. Houve maior mortalidade em pacientes com CCA extra-hepáticos.

**CONCLUSÃO:**

Fatores de risco como tabagismo, colecistectomia, cirrose, litíase intra-hepática e transplante devem ser considerados individualmente pelo médico assistente para realização de acompanhamento radiológico e descoberta incidental da neoplasia. O atraso no reconhecimento pelo médico assistente pode atrasar o diagnóstico, aumentando a mortalidade.

## INTRODUCTION

Cholangiocarcinoma (CCA) comprises a heterogeneous group of rare neoplasms originating in the epithelial cells of the bile ducts or from the peribiliary glands and hepatocytes, depending on the underlying liver disease and location^11,12,13,14^. It accounts for about 3% of carcinomas of the gastrointestinal tract^1^. CCA incidence is rare in developed countries (0.2-2 cases/100,000 population) but higher in Eastern countries where liver parasites are endemic^10^.

CCA cases are classified as intrahepatic (iCCA) when the neoplasm arises above the hepatic hilum and as extrahepatic (eCCA) when the neoplasm arises at the hepatic hilum (Klatskin tumor) or below it (distal CCA)^1,2^. iCCA cases represent about 15% of malignant neoplasms of the liver, ranking second in incidence after hepatocarcinomas^3^. eCCA has a similar incidence to iCCA^1,3^. In Brazil, the National Cancer Institute (INCA) does not collect data on bile duct cancer.

These neoplasms have diverse incidence rates in different regions of the planet. Nevertheless, the clinical picture is typical for the topography, infiltration pattern, and time of evolution^1,2,7,16^. The mean age at iCCA diagnosis is 55 years, with a slight male predominance. About 85% of patients have symptoms such as abdominal pain, jaundice, ascites, weight loss, fatigue, anorexia, nausea, and vomiting. A palpable mass is identified in the right abdominal quadrant in 5-30% of patients, associated with or without elevated serum levels of carcinoembryonic antigen (CEA) and cancer antigen 19-9 (CA19-9) and normal alpha fetoprotein (AFP) levels^1,3,7,10^. The diagnosis is made using computed tomography or magnetic resonance imaging, which exhibits a homogeneous lesion with irregular margins, intratumoral calcifications, and delayed contrast enhancement^1,2,3,16^.

iCCA^4,17,18^ can be further divided into small- and large-duct types. Large-duct iCCA commonly causes cholestasis or cholangitis, presenting as nodular or sclerosing periductal lesions with obliteration or stenosis. By contrast, small-duct iCCA becomes symptomatic only when the tumor reaches a considerable size, presenting as white or grayish masses that coalesce in advanced stages.

## METHODS

This study was approved by the Research Ethics Committee at Irmandade Santa Casa de Misericórdia, Porto Alegre, Brazil (protocol no. 5.807.740).

This analytical retrospective cross-sectional study selected CCA cases diagnosed at Irmandade Santa Casa de Misericórdia and sent them for biopsy examination at the hospital’s Pathology Laboratory in Porto Alegre between January 2016 and December 2021. Searches for CCA cases were conducted in the Pathox digital archive, and electronic medical records available in Tasy were evaluated, resulting in a sample of 96 CCA cases. All patients with an anatomopathological and/or immunohistochemical diagnosis of CCA were included. Patients whose diagnoses were suggestive of CCA but inconclusive were excluded. The following variables were collected from the electronic medical records and anatomopathological reports: sex, age, lesion size and topography, associated diseases, previous treatments, angiolymphatic invasion, perineural invasion, lymph node metastasis, resection margins, survival time (months), and treatment protocol.

Survival data were obtained from the date of death or the date of the last consultation available in the medical records. Laboratory results (bilirubin, CA19-9, and CEA) were also collected.

The classification of anatomical locations and tumor staging were based on radiological studies, combined with the findings reported in surgical descriptions and anatomopathological studies. Categorical variables were presented as absolute and relative frequencies. Quantitative variables were expressed as median and interquartile range (IQR), given their asymmetric distribution, as assessed by the Kolmogorov–Smirnov test.

Survival was analyzed using Kaplan–Meier curves, and hazard ratio estimates were obtained by Cox regression analysis. Results with a p < 0.05 were deemed significant. Analyses were performed using the SPSS software version 25.

## RESULTS

There was a 56% prevalence of CCA in women, with intrahepatic involvement in 55.4% and extrahepatic involvement in 44.6% of patients. Unifocality was observed in 85.6% of patients. Treatment consisted of surgery in 33.3%, chemotherapy in 32.1%, comfort measures in 19.8%, and a combination of therapies in the remaining patients. At the end of the study, the mortality rate was 81.6%.

The mean age of the patients was 63 years (26–89 years), with a mean tumor size of 5.5 cm (1.0–15.0 cm). The median survival time was 7 months (0 to >50).

CA19-9 was altered in 81% of patients, with a median value of 223 U/mL (3–40,000 U/mL). Laboratory results for aspartate aminotransferase/alanine aminotransferase (AST/ALT) were altered in 62.5% of patients and gamma-glutamyl transferase/alkaline phosphatase/bilirubin in 69.1% of patients.

Numerous previous diseases were identified in the medical records of the patients. Conditions with an increased prevalence in CCA patients compared with the same-age population included cholecystectomy, cirrhosis, intrahepatic lithiasis, transplantation, and viral hepatitis. The most common clinical feature was jaundice, associated with weight loss, abdominal pain, itching, nausea, and vomiting.

A total of 37 hepatic resections were performed, with compromised margins in 27%, positive lymph node status in 18%, angiolymphatic invasion in 37.8%, and perineural invasion in 45.9% of patients.

## DISCUSSION

eCCAs^1,3,6,10^ are commonly sporadic in individuals aged above 70 years and in the fourth and fifth decades of life when related to primary sclerosing cholangitis. The incidence rate^3,6^ is 0.53–2 cases per 100,000 population, being more common in Asian countries and in men with primary sclerosing cholangitis associated with inflammatory bowel disease.

Clinical features include jaundice, even in cases with a small tumor volume, given its obstructive effect, associated with or without pain in the right upper quadrant, malaise, weight loss, pruritus, anorexia, nausea, and vomiting. Additionally, eCCA overlaps with the diagnosis of cholangitis when it causes fever and chills^2,3,5,8,11^.

Useful examinations to determine the exact location of the neoplasm, possibly allowing early diagnosis, include transhepatic cholangiogram, cholangioresonance, endoscopic retrograde cholangiopancreatography (ERCP), endoscopic ultrasound, and positron emission tomography scan^16^. Generally, the best option is ERCP^11,16,24^.

It is reported that polypoid forms have a higher rate of early detection. Tumor obstruction may cause proximal ductal dilatation and periductal thickening, also showing contrast enhancement. Laboratory findings demonstrate elevation of the same markers (CA19-9 and CEA), which may be absent in 10% of cases. Diagnostic delay occurs in about 9% of cases, either due to an overlap or a misdiagnosis of cholecystitis, cholelithiasis, or cholangitis^9,25,27^.

Two types of precursor lesions^20,21^ are associated with the development of eCCA and large-duct iCCA: biliary intraepithelial neoplasm and intraductal papillary neoplasm. There are no precursor lesions^20,21^ defined for small-duct iCCA, suggesting its origin in progenitor liver cells or mature hepatocytes.

Some risk factors are simultaneously associated with the three subtypes, whereas others are specific to a given subtype. Caroli disease and choledochal cysts have a strong association with the three CCA subtypes^22^. Cirrhosis, nonalcoholic fatty liver disease, and hepatitis B have a stronger association with iCCA, whereas choledocholithiasis has a stronger correlation with eCCA^22^. In Western countries, primary sclerosing cholangitis is the main risk factor for CCA^23^; however, most cases do not have a well-established etiology.

Studies conducted in the last decade revealed an increase in the global incidence of CCA^3,5,12^. It is assumed that chronic inflammation of the bile ducts favors carcinogenesis by damaging DNA and altering the control of apoptosis and cell proliferation. The increase in iCCA cases can be attributed to the prevalence of cirrhosis and obesity in the population, as well as increased access to and the efficiency of diagnostic techniques^5,12^.

When radiological and immunohistochemical resources were not available, iCCA was often misdiagnosed as metastatic carcinoma. By contrast, there has been a decrease in the incidence of eCCA, probably due to the higher rates of cholecystectomies, which cause a decrease in gallstones, an important risk factor for gallbladder carcinomas and eCCA^2,3,5,12,15^.

The mean age at diagnosis, lesion size, survival rate, location, increase in CA19-9, and risk factors, such as cirrhosis, intrahepatic lithiasis, transplantation, and viral hepatitis, of the study sample were consistent with literature data^1,2,3,17^. A divergent finding was the predominance of women in this sample (55% versus 45%).

Risk factors such as smoking, dyslipidemia, and type 2 diabetes mellitus were not higher than expected for age and had no significant impact on pathophysiology, contrary to initial expectations. The lack of correlation with these risk factors could be attributed to incomplete medical records or to the fact that these factors were considered irrelevant to CCA diagnosis by the assisting teams.

Genetic findings for both iCCA and eCCA include alterations in TP53, KRAS, SMAD4, ARID1A, and GNAS^2^. TP53 mutation was observed in 50% of eCCA cases at the end of the carcinogenesis process^2^. KRAS mutations were considered initial events in 20-30% of cases^2^. MDM2 amplification occurs in 12% of perihilar CCAs but rarely in more distal ducts^2^. Other alterations identified in iCCA include IDH1/2, BRAF, and BAP1 mutations, as well as EGFR, PIK3CA, MET, and FGFR2 fusions. These findings allow molecular classification into inflammation and proliferation subclasses, according to the pattern of genetic alteration^1,2,25,26^.

The immunohistochemical profile of biliary neoplasms is relatively nonspecific, with frequent positivity for yytokeratin 7 (CK7), CK19, epithelial membrane antigen (EMA) mucin-1 (MUC1), and CA19-9, and the absence of a typical immunophenotype for other primary sites. Small-duct iCCA exhibited increased expression for CD56, PCR, N-cadherin, and IDH1/2 mutation, whereas large-duct iCCA showed increased expression for MUC5AC, MUC6, S100, TTF1, AGR2, MMP7, and KRAS mutation^2,5,8,18,19^.

The topographic classification of CCA can be challenging in advanced diseases when the neoplasm infiltrates both the parenchyma and the choledochum^3^. Comparison with previous literature data resulted in conflicting findings^3,12^, given the fact that the International Code of Diseases previously classified hilar tumors as iCCAs.

On histopathological examination^17,20^, eCCAs exhibit an adenocarcinoma generally of the pancreatobiliary type, characterized by small, irregular, well-formed, spaced glands, with frequent neurotropism and angiolymphatic invasion. Other histological types^17,20,28^ include the intestinal, foveolar, mucinous, signet ring, clear cell, pyloric gland, hepatoid, and micropapillary types. The adenocarcinomas are rarely epidermoid, adenosquamous, sarcomatoid, or undifferentiated.

The differential diagnosis of adenocarcinomas with reactive periductal glands represents a challenge, as it is not possible to differentiate them histologically or immunophenotypically from pancreatic ductal adenocarcinomas, requiring clinical and radiological correlation^2,5,6,10,11^.

The American Joint Committee on Cancer (AJCC) staging is performed based on topography (perihilar or distal). Large-duct iCCA occurs in larger intrahepatic bile ducts, near the hepatic hilum, and is similar to eCCA. Small-duct iCCA is usually located in the most peripheral liver parenchyma, with tumor mass formation and histological findings of a tubular, ductular pattern or strands of columnar to cuboidal cells with desmoplastic reaction, without mucin secretion. Large-duct iCCA shows macroscopic aspects of periductal infiltration associated with or without tumor mass formation, according to the time of evolution. Histological findings include a tubular or ductal pattern with desmoplastic reaction but associated with mucin production. This type may also exhibit the same rare histological types associated with eCCA. Both are classified as well, moderately, or poorly differentiated^1,2,3,27^.

In indicated cases, complete surgical resection and liver transplantation are treatment options for patients with small iCCAs, with the possibility of cure^11,25^. For advanced cases, chemotherapy and locoregional treatments, such as ablation, transarterial chemotherapy, radioembolization, and radiotherapy, have been shown to increase disease-free survival. Some randomized studies have suggested immunodirected therapies, such as checkpoint blockade immunotherapy, which had modest efficacy in CCA^11,22,26^.

eCCA prognosis^3,11,27^ is dependent on the stage of diagnosis. Resectable cases have survival rates of 20-30%, but those of unresectable cases are virtually null. Microscopic factors associated with worse prognosis include poor differentiation and vascular and perineural invasion^3,9,21^.

For iCCA, prognosis^3,10,27^ is generally guarded, with a small difference between resectable cases. Some of the microscopic characteristics related to a worse prognosis are vascular invasion, surgical margins compromised by the neoplasm, and advanced TNM (tumor, node, and metastasis) stage.

Small-duct types result in a longer survival than large-duct types, as the latter generally exhibits higher TNM stage and perineural invasion. In iCCA, polymerase chain reaction (PCR) expression is associated with a better prognosis, whereas EMA (MUC1) expression indicates a worse prognosis. Cirrhotic patients with iCCA have a similar prognosis to patients with hepatocarcinomas, and noncirrhotic patients have a worse prognosis. Unfortunately, the overall prognosis remains very bleak, with a 5-year mortality rate of 95%^4^.

In view of the severity of the diagnosis and the limited knowledge of the disease due to its rarity, this study sought to evaluate the prevalence, survival, and potential prognostic and therapeutic implications of CCA in a retrospective cohort of patients. Improved knowledge is expected to contribute to the early diagnosis and increased survival of these patients.

The limitations of this study are mainly related to the scarcity of information in the medical records and difficulties in the topographic classification of large tumors, not allowing adequate definition of the location (intra- or extrahepatic). Furthermore, it was difficult to compare the results with the literature because the International Code of Diseases previously described hilar tumors as iCCA (currently classified as eCCA).

## CONCLUSIONS

CCA, a malignant neoplasm of low incidence, is notable for its high lethality. Several risk factors contribute to its etiology, which is often unknown. Smoking and a history of cholecystectomy demonstrate a modest association, with a relative risk of up to twofold. Liver cirrhosis elevates the risk by up to 7-fold, and intrahepatic lithiasis and a history of liver transplantation are associated with substantial increases in risk, up to 4-fold and 25-fold, respectively. Individualized assessment of these risk factors is crucial in clinical practice, favoring the implementation of radiological surveillance for early detection.

The prognosis of eCCA is unfavorable, in accordance with previous literature reports. Symptom similarity with benign conditions such as cholelithiasis, cholecystitis, and cholangitis often delays diagnosis, highlighting the importance of raising awareness among health professionals about this clinical differentiation.

Effective communication between the attending staff and the pathology department is essential to ensure an accurate diagnosis. Often, crucial clinical information, such as serum levels of tumor markers (CA19-9, AFP), lesion size and location, history of previous neoplasms, and clinical hypotheses, is not properly shared, compromising the diagnosis and, therefore, the therapeutic approach.

The disparity in survival rates between patients with hypothyroidism and the general population is remarkable. Although some studies suggested that hypothyroid patients may experience increased survival, the underlying nature of this association remains under investigation.

Finally, this study observed a predominance of female patients with CCA, suggestive of a regional epidemiological peculiarity. Additional studies are needed to confirm the nature and extent of this association.
